# The economic burden of overseas medical treatment: a cross sectional study of Maldivian medical travelers

**DOI:** 10.1186/s12913-015-1054-2

**Published:** 2015-09-26

**Authors:** Mariyam Suzana, Anne Mills, Viroj Tangcharoensathien, Virasakdi Chongsuvivatwong

**Affiliations:** Epidemiology Unit, Faculty of Medicine, Prince of Songkla University, Songkla, 90110 Thailand; Faculty of Public Health and Policy, London School of Hygiene and Tropical Medicine, Keppel Street, WC1E7HT London, UK; International Health Policy Program, Ministry of Public Health, Tiwanon Road, Nonthaburi, 11000 Thailand

**Keywords:** Medical treatment overseas, Maldives, Health finance, Medical tourism

## Abstract

**Background:**

Access to tertiary care is a problem common to many small states, especially island ones. Although medical treatment overseas (MTO) may result in cost savings to high income countries, it can be a relatively high cost for low and middle income source countries. The purpose of this study was to estimate the costs of overseas medical treatment incurred by the households of medical travelers from Maldives and assess the burden of medical treatment overseas on the government and on households.

**Methods:**

A survey was conducted of inbound Maldivian medical travelers who traveled during the period June – December 2013. Participants were stratified by the source of funds used for treatment abroad. Three hundred and forty four government-subsidized and 471 privately funded Maldivians were interviewed. Self-reported data on the utilization and expenses incurred during the last visit abroad, including both expenses covered by the government and borne by the household, were collected using a researcher administered structured questionnaire.

**Results:**

The median per capita total cost of a medical travel episode amounted to $1,470. Forty eight percent of the cost was spent on travel. Twenty six percent was spent on direct medical costs, which were markedly higher among patients subsidized by the government than self-funded patients (*p* = <0.001). The two highest areas of spending for public funds were neoplasms and diseases of the circulatory system in contrast to diseases of the musculoskeletal system and nervous system for privately funded patients. Medical treatment overseas imposed a considerable burden on households as 43 % of the households of medical travelers suffered from catastrophic health spending. Annually, an estimated $68.9 million was spent to obtain treatment for Maldivians in overseas health facilities ($204 per capita), representing 4.8 % of the country’s GDP.

**Conclusions:**

Overseas medical treatment represents a substantial economic burden to the Maldives in terms of lost consumer spending in the local economy and catastrophic health spending by households. Geographical inequality in access to public funds for MTO and the disproportionate travel cost borne by travelers from rural areas need to be addressed in the existing Universal Health Care programme to minimize the burden of MTO. Increased investment to create more capacity in the domestic health infrastructure either through government, private or by foreign direct investment can help divert the outflow on MTO.

## Background

Globalization and technological advancements have facilitated cross country trade in health services, especially in the mobility of patients to seek medical treatment overseas (MTO). The medical tourism climate survey of 2013, carried out in 400 health facilities in 77 countries, explains this phenomena: 60 % of the facilities experienced growth in the number of international patients in 2012, and 80 % expected the growth of international patient numbers over the next 12 months [1]. In Asia, 10 million medical travelers are expected to seek treatment in a few Asian destinations by 2015, doubling the market size of 2011 [2].

Medical treatment overseas, categorized as mode 2 of the General Agreement on Trade in Services (GATS), is defined as the supply of health service in the territory of one member to the consumers of any other member [3]. Organized MTO may result in cost savings to high income countries sending their patients to use services abroad if the cost is lower than in-country services. However, it can be a relatively high cost for low and middle income source countries. Indonesia produced 1 million medical tourists per year in 2007 and 2008, spending $1 to $1.5 billion abroad [4], while Samoans spent $2.88 million in 2007, [5] and $1.5 million was expended on overseas medical providers in 2009 by Seychelles government [6].

Access to tertiary care is a problem common to many small states, especially island ones. This study was undertaken in the Maldives, a small island state situated in the Indian Ocean. The Maldives is one of the 53 small island developing states (SIDS) [7], characterized by their narrow economic base, high production costs, shortage of skilled labor, heavy dependence on trade and foreign aid. It has an ethnically homogenous population of 336,224 [8] dispersed over 198 very small islands. Only 4 islands have a population exceeding 5000, and 72 of the islands are populated by less than 500 people [9]. The Maldivian health system is customized to fit its unique geography. One primary health facility is located on each inhabited island. A group of 10 islands on average makes up one atoll, and one atoll hospital is located in each of the 21 atolls providing secondary health care. Six regional hospitals serve up to 3 to 4 atolls each. Tertiary care is available in the capital city, Male’. The Indian medical tourism industry outlook 2015 showed that Maldives was one of the top importers of health care from India [10].

The Maldives ranks as a top achiever in health indicators, and has the highest total spending on health in South East Asia at US$ 558 per capita or 8.5 % of the GDP in 2011 [11]. Financed through the government budget, universal health care was introduced in the Maldives from 2012, with free health care at point of service. Government subsidy for MTO is available from contracted providers only and requires a recommendation from a public sector specialist doctor. It is limited to direct medical costs and the airfare of the patient and one caretaker which are directly paid to airlines and providers by the government. Expenses not covered by the UHC program are paid either through the private insurance market or from out of pocket. In 2011, the national health accounts of the Maldives estimated household out of pocket expenditure on MTO at $42.5 million which was $133 per capita [12]. A survey on Maldivian travel abroad (MTA 2013) conducted by the Central Bank of Maldives showed that one out of two Maldivians traveled overseas for various purposes in 2013 and spent $70 million on medical travel alone [13].

The focus of many of the existing studies on MTO is on the supply side of the industry. Empirical findings on the demand for MTO and its effects on the resource constrained economy are essential to guide better policy responses. The key features that differentiate the setting from other countries facing similar problems are its remoteness and smallness that limit the opportunities for economies of scale and competition, which increases the cost of inputs for the provision of health care. This study aimed to estimate the current costs of medical treatment overseas, and assessed the burden of MTO both to the government and households. It contributes to evidence-based policy responses to address health system issues common to small and remote countries in a sustainable way.

## Methods

### Study design

A survey of Maldivians who traveled for medical treatment during the period June – December 2013 was conducted. A sample of 342 government subsidized and 418 privately funded travelers was needed in order to estimate costs with a precision of at most 5 % difference from the average actual costs in each group and with the assumption of a 30 % non-response rate.

### Participants

Participants were recruited from three sources and stratified by the type of funds used to pay for treatment abroad. The first source used was the national database of patients who had traveled under government financial assistance. The 2013 database is maintained by the ‘Aasandha’ universal coverage program covering patients from all the 21 administrative atolls of Maldives. Out of 2556 who traveled during the study period, 344 were randomly selected and a telephone interview was conducted. This data source consisted of government subsidized travelers only.

Secondly, the three international airports of Maldives located in the North, Center and South (Haadhaal atoll, Kaafu atoll and Seenu atoll respectively) were surveyed to reach privately funded travelers. All international flights from all destinations were eligible. As the North and South airports operated only one international flight per week, the Central airport was also surveyed on one random day of the week. Face to face interviews were conducted with a consecutive sample of 335 inbound travelers during August to December 2013.

As a third source, the regional level health facilities in the aforementioned three atolls were contacted. With the assistance of the management of the health facility, privately funded travelers were identified from lists of referrals made abroad. Telephone interviews were conducted with 136 available informants. Hence a total of 471 self-funded patients were interviewed.

A total of 344 government subsidized and 471 privately funded subjects were interviewed using a common questionnaire among informants from all the three data sources to acquire demographic and household characteristics, utilization and cost data. Overlapping informants were self-identified and repetitive collection from the same person was avoided. Proxy respondents who were adult relatives or caretakers were used for elderly, young or very sick patients.

**Utilization data on MTO** included the country and hospital of treatment, length of stay for the last visit, number of accompanying persons, the number of MTO visits made per year and primary diagnosis for which treatment was given. Diseases were coded under the 22 major groupings of ICD 10, version 2010 [14]. Ailments or descriptions that did not fall under any major disease group were coded under “Factors influencing health status and contact with health services” (Z00-Z99 of ICD10). This group (169 observations) was omitted from the analysis of disease profile following WHO advice not to use this group for comparisons [15].

### Costs of MTO

Cost data was collected on direct medical expenditures, indirect expenditures and productivity loss. Direct medical expenditures included outpatient, inpatient, procedure or surgery, diagnostics, medicines, room or ward charge, and other hospital costs. As responses were based on recall, expenditure breakdowns for medical care were not available in most interviews. Therefore, only the total costs for medical care, available for all the subjects, were used in the analysis. Indirect expenditures consisted of travel costs, agent or dealer costs, entertainment costs, food, lodging, and other costs. Productivity loss was calculated by multiplying the number of hours lost per day by the number of days spent overseas and by the income per day as reported by the participant. All cost categories for the MTO visit were collected by government and household contributions.

### Household expenditures

In addition to MTO costs, monthly out of pocket expenditure on health, food and other expenses was collected for the purpose of calculating the catastrophic expenditure on health. Catastrophic health expenditure was derived as the proportion of health spending out of total household expenditure [16]. The incidence of catastrophic payments was defined as payments in excess of a threshold budget share and the intensity of catastrophic expenditure was measured by the payment in excess of the threshold, averaged over all households exceeding that threshold [16]. The threshold level was set at 10 % of total household expenditure.

### Economic costs

**Fig. 1:**
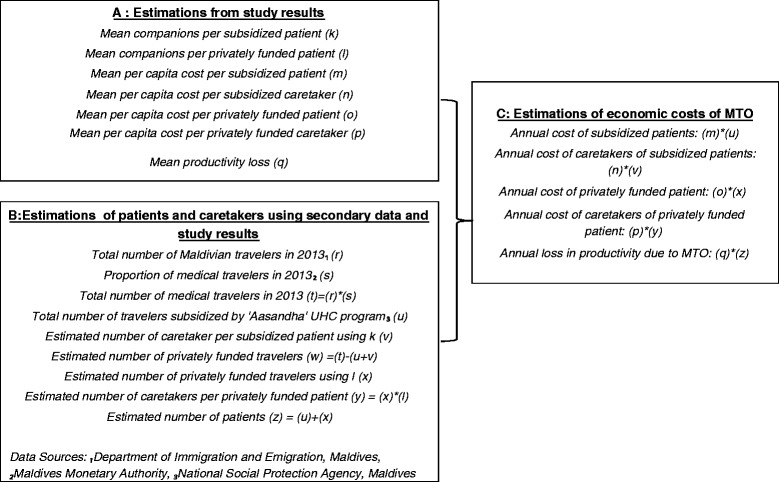
Methodology for the extrapolation of Economic costs of MTO using study results and secondary data sources

The total economic cost of MTO to the country was calculated by extrapolating the results from the study samples to the population estimates of patients and caretakers travelling for medical purposes obtained from secondary data. Figure 1 shows how the economic costs for MTO were derived. Estimations in block C are the products of estimations in block A and B.

### Statistical analysis

The household was the unit of analysis in this study and the societal perspective was adopted. For analytical purposes, the following continuous variables were collapsed into categorical data: age, household income, expenditure, length of stay and household size. Based on the location of the island, the residential island of the patient was grouped into three regions. Derived variables for the analysis include catastrophic expenditure and total cost per traveler per visit. Per capita cost was calculated separately for the patient and caretaker taking into account that caretakers do not bear medical costs. Productivity loss was excluded in the estimation of per capita cost as it was not a cost but a loss. Frequencies and median (inter quartile range) were used to describe categorical and continuous data respectively. Non-parametric tests for trends were used, as the main outcome measures (costs) were highly skewed. The standard errors of the estimates of economic costs of MTO were derived by the bootstrap technique which uses a large number of randomly drawn resamples of size ‘*n’* from the original sample to estimate the standard error. The level of significance was set at *p* < 0.05, and data analysis was conducted using the open source R software, version 3.1.0 [17].

### Ethical approval

The research was undertaken as a partial fulfillment of the PhD in epidemiology program of the Prince of Songkla University (PSU). Hence, ethical approval was obtained from the Ethics Committee of PSU and the Research Ethics Committee of the Ministry of Health of Maldives. Administrative approval was sought from the Ministry of Transport and Communications to conduct the airport survey in the three regional airports. The translated version of the questionnaire was validated in written format by the Ministry of Education of Maldives.

## Results

### General characteristics

**Fig. 2:**
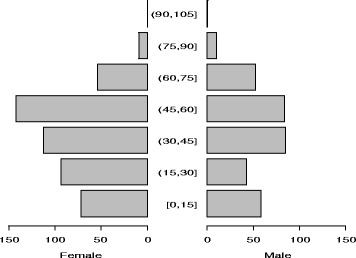
Population pyramid of medical travelers by age and sex

Out of the 815 medical travelers interviewed, 42 % were subsidized by the government. Females in the adult age group made up a considerable share of the sample, (Fig. 2). Table 1 shows the demographic and utilization patterns among government-subsidized and privately funded patients. Government subsidized patients were more likely to reside in the central region, be unemployed and be in the adult and the elderly age groups (*p* = <0.001). Subsidized travelers had an equal distribution by gender, while a higher proportion of females (66.5 %) were among the privately funded patients. The two groups did not differ in their household size (*p* = 0.3) or income of the past month (*p* = 0.5).

**Table 1:** Demographic characteristics and utilization pattern among two types of medical travelers, 2013

	Government subsidized travelers	Privately funded travelers	Total	*P* value
	*N* (%)	*N* (%)	*N* (%)	
Total	344 (42.2)	471 (57.8)	815 (100)	
Gender				<0.001
Female	169 (49.1)	313 (66.5)	482 (59.1)	
Male	175 (50.9)	158 (33.5)	333 (40.9)	
Age group				<0.001
Children <=9 yrs	53 (15.4)	46 (9.8)	99 (12.1)	
Adolescents 10–19 yrs	24 (7)	34 (7.2)	58 (7.1)	
Youth 20–29 yrs	30 (8.7)	64 (13.6)	94 (11.5)	
Adult 30–59 yrs	163 (47.4)	266 (56.5)	429 (52.6)	
Elderly >=60 yrs	74 (21.5)	61 (13)	135 (16.6)	
Region of residence				<0.001
North	86 (25)	159 (33.8)	245 (30.1)	
Central	190 (55.2)	176 (37.4)	366 (44.9)	
South	68 (19.8)	136 (28.9)	204 (25)	
Occupation of the patient				0.007
Civil servant	52 (15.1)	119 (25.3)	171 (21)	
Private sector	35 (10.2)	42 (8.9)	77 (9.4)	
Own business	77 (22.4)	88 (18.7)	165 (20.2)	
Not employed	136 (39.5)	156 (33.1)	292 (35.8)	
Other	44 (12.8)	66 (14)	110 (13.5)	
Household size				0.362
Small (<5members)	201 (58.4)	277 (58.8)	478 (58.7)	
Medium (6–10 members)	100 (29.1)	149 (31.6)	249 (30.6)	
Large (>10 members)	43 (12.5)	45 (9.6)	88 (10.8)	
Household income in the past month^a^				0.499
Poorest 20 %	81 (23.5)	91 (19.3)	172 (21.1)	
2nd quintile	65 (18.9)	89 (18.9)	154 (18.9)	
3rd quintile	93 (27)	134 (28.5)	227 (27.9)	
4th quintile	59 (17.2)	78 (16.6)	137 (16.8)	
Richest 20 %	46 (13.4)	79 (16.8)	125 (15.3)	
Reason for seeking treatment abroad				<0.001
Quality of care	39 (11.3)	185 (39.3)	224 (27.5)	
Unavailability of service	234 (68)	126 (26.8)	360 (44.2)	
Continuity of care	4 (1.2)	23 (4.9)	27 (3.3)	
Better prices	2 (0.6)	19 (4)	21 (2.6)	
Long waiting time	8 (2.3)	18 (3.8)	26 (3.2)	
Other	57 (16.6)	100 (21.2)	157 (19.3)	
Country of treatment				0.087
India	239 (69.5)	305 (64.8)	544 (66.7)	
Sri Lanka	105 (30.5)	160 (34)	265 (32.5)	
Thailand	0 (0)	5 (1.1)	5 (0.6)	
Italy	0 (0)	1 (0.2)	1 (0.1)	
Length of stay				<0.001
<=1 week	53 (15.4)	100 (21.2)	153 (18.8)	
2 weeks	95 (27.6)	180 (38.2)	275 (33.7)	
3 weeks	99 (28.8)	116 (24.6)	215 (26.4)	
1 month	18 (5.2)	30 (6.4)	48 (5.9)	
>1 month	79 (23)	45 (9.6)	124 (15.2)	

^a^Household income quintiles are based on the income of the study subjects

### Utilization pattern

Indian and Sri Lankan hospitals were the major destinations (98.1 %). Length of stay abroad and reasons for seeking medical treatment abroad differed significantly between the two groups. Privately funded patients were more likely to state that they went in search of better quality care, while government subsidized patients went abroad mainly because the required treatment was not available in the country. Government subsidized patients stayed overseas longer (*p* = <0.001). On average a subsidized medical traveler was accompanied by 2.3 persons, while 3.3 persons accompanied a privately funded patient.

### Disease profiles

Table 2 displays the disease groups for which overseas treatment was most sought by the two groups of medical travelers. The disease groups differed by age, residential region of the patient and by the type of financial coverage (details not displayed). A higher proportion of patients subsidized by the government were treated for neoplasms and diseases of the circulatory system (86.3 % and 57.3 %) in contrast to diseases of the musculoskeletal system and nervous system (84.6 % and 66.7 %) among privately funded travelers. No significant difference in socio economic status was observed among the patients suffering from different diseases (*p*-value = 0.5).

**Table 2:** Distribution of the ten disease groups for which overseas treatment was most sought by type of finance

Disease Groups (ICD code)	Number	Subsidized %	Privately funded %
Circulatory (I00–I99)	82	57.3	42.7
Nervous (G00–G99)	69	33.3	66.7
Musculoskeletal (M00–M99)	65	15.4	84.6
Genitourinary (N00–N99)	62	54.8	45.2
Eye (H00–H59)	51	54.9	45.1
Neoplasms (C00–D48)	51	86.3	13.7
Symptoms & signs (R00–R99)	49	28.6	71.4
Injuries (S00–T98)	43	34.9	65.1
Digestive (K00–K93)	35	40.0	60.0
Endocrine (E00–E90)	35	22.9	77.1

Table 3 shows the major disease groups with the highest treatment costs. Although the median medical expenditures were highest for accidents and trauma, the number of cases in this disease group was small resulting in relatively lower total costs. Diseases of the circulatory system, neoplasms and diseases of the nervous system were more common and hence had the highest proportion of medical expenditure among all travelers.

**Table 3:** Selected disease groups with the highest treatment costs

Disease group (ICD 10)	No of patients *N*	Median hospitalization price paid $	Total hospitalization cost $
External causes of morbidity & mortality	8	2,420.00	23,977.00
Congenital malformations	9	985.00	12,959.00
Neoplasms	51	890.00	108,769.00
Pregnancy, childbirth & puerperium	3	833.00	2,846.00
Diseases of the blood	7	800.00	29,515.00
Diseases of digestive system	35	700.00	44,004.00
Diseases of the circulatory system	82	691.00	181,215.00
Diseases of genitourinary system	62	603.00	52,151.00
Diseases of nervous system	69	593.00	112,048.00
Diseases of the respiratory system	25	500.00	50,811.00

### Direct medical expenditures

Table 4 shows the expenditures incurred per visit overseas for medical treatment. On average, 26 % of the total cost of treatment abroad was consumed by direct medical costs, which were markedly higher among patients funded by the government (*p* value < 0.001). Other determinants of high cost included being male and coming from the southern region of the country. Table 5 shows the breakdown of expenditures among the subsidized travelers. The government subsidy for MTO provided partial coverage for direct medical costs and travel costs, and accounted for 38 % of the total cost of the subsidized traveler. The median medical expenditure of subsidized travelers was on average 67 % higher than non-subsidized patients.

**Table 4:** Selected expenditures per visit overseas

	Total		Government subsidized	Privately funded	*P* value
	Mean share of cost %	Median (IQR)$	Median (IQR) $	Median (IQR) $	
No of patients		815	344	471	
Direct medical costs	26.0 %	500 (181.5–1150)	825 (300–2085)	300 (147.5–720.5)	<0.001
Indirect costs:					
Travel	48.0 %	1157 (781–1777.5)	1197 (817.2–1774.2)	1133 (739.5–1804.5)	0.14
Entertainment	8.0 %	100 (0–300)	34.5 (0–200)	142 (0–400)	<0.001
Lodging	8.0 %	140 (50–300)	150 (67–300)	127 (47–300)	0.053
Food	6.0 %	105 (50–250)	140 (56–300)	100 (46–210)	0.001
^a^Total cost (Patients and caretakers inclusive)		2608 (1749.5–3914)	2984 (1953.2–5041.5)	2355 (1564–3520.5)	<0.001
^a^Total cost per capita (patient)		1470.8 (888.2–2353.2)	1905.1 (1254.4–3554.4)	1147.3 (709.6–1783)	<0.001

Costs that constituted a share of <1 % were omitted, ^a^excludes productivity loss, *IQR:* inter-quartile range

**Table 5:** Breakdown of expenditures among subsidized travelers (*n* = 344)

	Subsidy $	Out of pocket $
	Median (IQR)	Median (IQR)
Direct medical costs	500 (106.25–1462.5)	56.5 (0–500)
Indirect costs:		
Travel in home country	0 (0–0)	81.5 (8–437.25)
Air fare	336 (200–419.25 )	400 (336–672)
Travel in destination country	-	91 (35–200)
Entertainment	-	34.5 (0–200)
Lodging	-	150 (67–300)
Food	-	140 (56–300)
Visa	-	0 (0–0)
Foreign exchange costs	-	0 (0–0)
Other costs	-	0 (0–0)
Productivity loss	-	0 (0–202)
Total cost	909(493.25–1805)	1797 (1137.5–3092.25)
Average share of total cost	38 %	62 %

IQR: inter- quartile range

### Indirect expenditures

Non-medical expenditures followed the same pattern with all expenses being slightly higher among government subsidized patients, except for entertainment costs which were considerably lower (*p* = <0.001). Patients from the central region paid comparatively lower travel costs than patients living in other regions (*p* = <0.001). Food and lodging expenses overseas did not differ by demographic or socio-economic status of the patient. Travel cost absorbed 48 % of the total cost with no significant difference between the groups. Excluding airfare, expenditure on travel in the home country was on average $280 compared to $155 in the destination country (details not shown).

The median per capita total cost of a medical travel episode was $1,470. Unforeseen expenditures such as agent fees, foreign exchange costs, and transfer of money from home country due to unpredicted expenses was very low due to the small number of patients who incurred these expenses. A few patients paid as much as $1,091 in mortuary fees or purchase of land for burial and as much as $818 for agent fees during their visit abroad (details not shown). These hidden costs add to the financial burden of medical treatment overseas.

### Catastrophic health expenditure

**Fig. 3:**
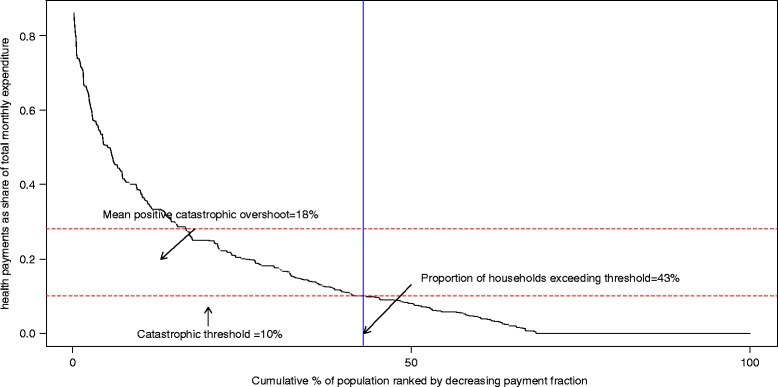
Incidence and intensity of catastrophic health spending among medical travelers

Health expenditures in the sample population increased proportionately with increasing total household expenditures (*p* = <0.001). Figure 3 shows the incidence and intensity of catastrophic payments on health among medical travelers. 43 % of the households experienced catastrophic health expenditure, measured by the households which spent more than 10 % of their monthly expenditure on health care. On average these households spent 28 % (10 % + 18 % mean positive overshoot) of their household resources. Between the households with and without catastrophic expenditure, there was no significant difference in household size, household income or the type of finance that the patient used. However, regional differences were observed (Table 6). Most households that were protected from catastrophic health spending were located in the Central region, while catastrophic health spending was highest among households in the South (*p* < 0.001). Most common diseases among households that incurred catastrophic health expenditures were diseases of the circulatory system (11.1 %) and diseases of genitourinary system (9.1 %).

**Table 6:** Catastrophic health expenditure by type of finance, residential area, household size and household income of the patient

	Households with health spending <10 % of total monthly expenditures	Households with health spending >10 % of total monthly expenditures	Total	*P* value
	*N* (%)	*N* (%)	*N*	
Total	465 (57.1)	350 (42.9)	815	
Type of finance				0.7
Government subsidized	199 (57.8)	145 (42.2)	344	
Private finance	266 (56.5)	205 (43.5)	471	
Region of residence				<0.001
North	142 (58)	103 (42)	245	
Central	247 (67.5)	119 (32.5)	366	
South	76 (37.3)	128 (62.7)	204	
Household size				0.4
Small (<5 members)	273 (57.1)	205 (42.9)	478	
Medium (6–10 members)	147 (59)	102 (41)	249	
Large (>10 members)	45 (51.1)	43 (48.9)	88	
Household income				0.1
Poorest 20 %	86 (50)	86 (50)	172	
2nd quintile	87 (56.5)	67 (43.5)	154	
3rd quintile	127 (55.9)	100 (44.1)	227	
4th quintile	84 (61.3)	53 (38.7)	137	
Richest 20 %	81 (64.8)	44 (35.2)	125	

### Economic costs

Table 7 shows the economic costs of medical treatment overseas to the Maldives derived from Fig. 1. Through extrapolation of study results, it was estimated that $68.9 million was spent in seeking medical treatment overseas per year by Maldivians which is $204 per capita. Using 2013 estimates of GDP [18], annual expenditure on MTO by medical travelers represents 4.8 % of the country’s GDP. As the government subsidy covered 38 % of the costs borne by subsidized travelers, it is estimated that the government disburses $5.5million per year on MTO (details not displayed). This accounts for 8 % of the total expenditures on MTO per year.

**Table 7:** Economic cost of medical treatment overseas

	Median per capita cost per visit (IQR) $	Est no of medical visits 2013	Annual cost per year (^a^se) $	% of GDP 2013 (^a^se)
	(a)	(b)	(c) = (a × b)	
Government subsidized (*n* = 344)				
medical travelers	1905.08 (1254.4,3554.4)	^b^3546	6,755,426 (851,408.3)	0.47 (0.11)
caretakers (mean = 2.3)	965.25 (679.8,1330.5)	8156	7,872,579 (387,083.3)	0.55 (0.08)
Private funded (*n* = 471)				
medical travelers	1147.33 (709.6,1783)	15462	17,740,068 (1,595,637)	1.24 (0.08)
caretakers (mean = 3.3)	716.21 (470.6,1023)	51026	36,544,821 (1,656,017)	2.55 (0.07)
Productivity loss (*n* = 815)	0 (0,156.5)		0 (57.23)	0 (0)
Total		78190	68,912,894 (5,586,963)	4.81 (0.07)

(a) Costs from Study results, (b) population estimates from secondary data and study results, (c) extrapolations using (a) and (b)

GDP 2013 = $ 1,431 m, ^b^number of visits subsidized by UC program, ^a^Bootstrap estimations

## Discussion

Using data on both government subsidized patients and privately funded patients, this study has estimated the costs of medical treatment overseas to households and the economy of Maldives. When the health system cannot offer all services required by the population, it results in patients seeking care overseas, often with high medical bills. It was found that catastrophic health spending was unavoidable to many medical travelers, despite government subsidy, and MTO places a heavy financial burden on the economy of the source country.

Disease profile among travelers in this study differed from findings in the literature. Diseases of the circulatory system and nervous system were found to be the most common among Maldivian patients while medical travelers from high income countries sought low priority health services. US patients in Mexico sought dental visits mostly [19], Canadian patients sought diagnostic procedures [20] and UK patients went overseas for operations of the skin and its associated structures [21]. This may be explained by the heterogeneous nature of the medical tourists and the variations of health systems across countries.

The choice of Indian and Sri Lankan hospitals sought by 98 % of the travelers can be explained by proximity and familiarity. Patients from high income countries such as USA, UK, Canada and middle income countries such as Nigeria preferred India mainly for affordable prices and to avoid long waiting times [20, 22]. The fact that patients in this study sought services that were not available in the country and for quality of care deviated from the norm among high and middle income countries. It follows the characteristics of the poorer economies, where inadequate health infrastructures exist [23].

This study indicates that the government subsidy is a useful instrument for promoting access to health services not available in the country. The government has targeted vulnerable populations consisting of the elderly, and those with lengthy, costly diseases, and there were no gender or socio-economic differences amongst its beneficiaries. The results add to evidence from Thailand [24], Turkey [25] and Mexico [26] which showed that universal health care promotes equality and access to health services.

Government contribution to the MTO cost burden was found to be low. Public subsidy was estimated to be 8 % of the annual expenditure on MTO. The fact that people have chosen to opt out of the publicly funded system underlines critical shortfalls in the existing system. Geographical inequality in access to public subsidy for MTO and the availability of private insurance for MTO may contribute to inequities in access to MTO. The literature shows that the existence of a parallel private health insurance market that enables affordability of quality care [27] may push people to opt out of the public health system. A survey on medical travelers from 27 countries showed that 70 % of the travelers expected the costs of overseas treatment to be reimbursed by their health authorities [28]. Increasing accessibility to government subsidy for medical travel is important especially when the local health system is unable to cater to the needs of the people.

Travel costs absorbed the largest share of total cost of care overseas and out of pocket expense on travel in the home country was higher than in the destination countries. In addition, rural areas were found to bear a disproportionate travel burden. Many studies have so far established that transport costs have serious implications for the accessibility of health care for the people in need [29–31]. An inverse correlation between medical tourism travel barriers and willingness to use medical travel has also been identified (correlation coefficient = −0.320, *p* < 0.001) [32]. If purchase of overseas treatment is to be continued, a mechanism to reduce travel costs for medical travelers is needed.

Medical costs were found to be the second major expense in a medical travel episode and medical costs for privately funded patients were half that of the subsidized patients. The comparatively higher medical costs of government subsidized patients in this study may be partly due to targeting of the sick and old and partly due to inefficiencies in purchasing. The use of government to government bilateral diplomacy by the UK [33], strategic purchasing methods [34], foreign direct investments in Cambodia, Laos and Vietnam [35], telemedicine in Bhutan [36] and technology assessments by Taiwan [37] are a few of the strategies that have been used to contain costs in the publicly funded health systems.

This study revealed that households bear a heavy burden of MTO. 43 % of the households where medical travelers lived experienced catastrophic health expenditure. Catastrophic spending was highest among travelers from the Southern region where the proportion of travelers who accessed public subsidy for MTO was lowest. There have been no previous studies that measured catastrophic health spending due to treatment overseas. The geographical disparity in catastrophic health spending suggests a regional imbalance in access to public funds. The association between public financing of health care and reduced incidence of catastrophic health spending has been well established [24, 38, 39], and findings from this study adds to this literature on the importance of broadening access to public funds. When expanding coverage against a backdrop of geographical inequality and high catastrophic health spending, the disadvantaged groups seeking MTO and the high priority services for MTO need to be identified and targeted [40].

Similar to the high annual spending on overseas treatment observed from this study ($68.9 million per year), Bangladeshi medical tourists spent US$30 million in India alone during 1998, [41] while Nigerians spent twenty billion US dollars per year on medical travel [42] and UK patients spent $180 million in 2010 [21]. However, the large populations of these countries would yield low economic impact to the country’s economy in contrast to the $204 per capita estimated to be spent by Maldivians on medical treatment overseas. Our estimation on annual expenditure on MTO ($68.9 m) was similar to findings from the survey on Maldivian travel abroad (MTA 2013) which estimated $70 million as annual MTO expenditure [13]. The slight difference is due to methodological differences. While the MTA was conducted at a single international airport for one week among all types of travelers, our survey focused specifically on medical travelers from three international airports over a period of five months.

### Strengths and limitations

While this study covered the different types of costs using a combination of data sources which give better insight, the data were based on samples from only five months and may have suffered from under reporting due to recall errors. The study period was an intensely politicized period with presidential and parliamentary elections. This may have led to under reporting of variables such as productivity loss and over reporting of other variables such as hospitalization and travel expenses paid by the government. Since data on private insurance support was not collected, out-of-pocket expenditure might have been exaggerated but private insurance coverage is not expected to be high in the Maldives. As this study had limited data on the reasons people who paid privately chose to do so, an in depth qualitative study could explore the reasons for MTO in greater depth.

## Conclusions

Medical treatment overseas represents a substantial economic burden to the Maldives in terms of lost consumer spending in the local economy and catastrophic health spending by households. Unlike medical travelers from high income countries, Maldivians sought middle to high priority health services for which the existing benefit package needs to be prioritized. The medical cost of a subsidized traveler was comparatively higher than that of a privately funded traveler suggesting opportunities to minimize costs by increasing the efficiency with which healthcare is purchased from overseas health facilities. Geographical inequality in access to public funds for MTO and the disproportionate burden of travel cost in the home country borne by MTO travelers from rural areas need to be addressed in order to minimize the burden of MTO. While the Maldivian UHC program is financed through the government budget, the outflow on MTO represents a considerable diversion of public funds to overseas facilities. Increased investment to create more capacity in the domestic health infrastructure either through government, private or by foreign direct investment can help divert the outflow on MTO.

### What is already known on this topic

- Treatment overseas usually focuses on the term ‘medical tourism’ which targets people who willingly travel in search of quality care or better prices.
- The magnitude, effects, attitudes and costs of medical tourism have been documented, but mostly among well-established health systems.
- Cost estimations and cost comparisons of treatment procedures in destination countries showed high levels of economic burden to source countries. Focus is however mostly on the supply side of the industry.

### What this study adds

- The focus of this study is travelers having limited choice in their home country because tertiary care is neither available nor of good quality
- It demonstrates that MTO has a negative financial impact at the household level and on the economy in resource constrained health systems.
- It gives the demand side perspective of MTO.

## Abbreviations

GATS: General agreement on trade of services
GDP: Gross domestic product
ICD: International classification of diseases
IQR: Inter quartile range
MTO: Medical treatment overseas
MTA: Maldivians traveling abroad survey
MMA: Maldives monetary authority
NHA: National health accounts
OOP: Out of pocket spending
THE: Total health expenditure
UHC: Universal health care
$: United States Dollar
